# Laryngeal Giant Cell Tumor: A Case Report and Review of the Literature

**DOI:** 10.1155/2014/503497

**Published:** 2014-05-15

**Authors:** Jumpei Nota, Yoshihisa Okochi, Futoshi Watanabe, Tadahiko Saiki

**Affiliations:** Department of Otorhinolaryngology, Steel Memorial Hirohata Hospital, 3-1 Yumesaki-cho, Hirohata-ku, Himeji-shi, Hyogo 671-1122, Japan

## Abstract

Giant cell tumor (GCT) is a benign neoplasm arising most commonly in the long bones. GCTs of the larynx (GCTL) are relatively rare, and only individual case reports are documented in the literature. Patients with such tumors may present with hoarseness and anterior neck swelling. We present a 59-year-old man with hoarseness and enlarging anterior neck mass for 3 months. A fiberscopy revealed a submucosal swelling of the left subglottic trachea. Computed tomography and magnetic resonance imaging of the larynx demonstrated a large, well-defined, inhomogeneous enhancing mass at the left thyroid cartilage, which was obstructed entirely. The anterior neck mass was biopsied for histopathological analysis, which showed multinodularity with intervening vascularized connective tissues. The mass was made up of mononuclear cells and distributed multinucleated giant cells. The mitotic activity of the mononuclear cells was as high as 6 per 10 high-power fields. Pathologic consultation resulted in a diagnosis of giant cell tumor. The patient underwent total laryngectomy and, postoperatively, he did well without recurrence or metastasis for two and a half years.

## 1. Introduction

Giant cell tumor (GCT) is classified as a benign bone tumor, and it represents 4 to 9.5% of all bone tumors and 20% of all benign bone tumors [1]. It occurs in 20- to 45-year-old individuals, with a slightly higher predominance in women [2]. The tumor is frequently identified at the distal femur, proximal tibia, distal radius, proximal humerus, and sacrum [3]. Primary giant cell tumors of the larynx (GCTL) are very rare. Our review of the literature discovered 31 cases of GCTL reported since Wessely presented the first case in 1940 [4]. Herein, we report a new case of GCTL and present a concise review of the literature.

## 2. Case Presentation

**Figure 1:**
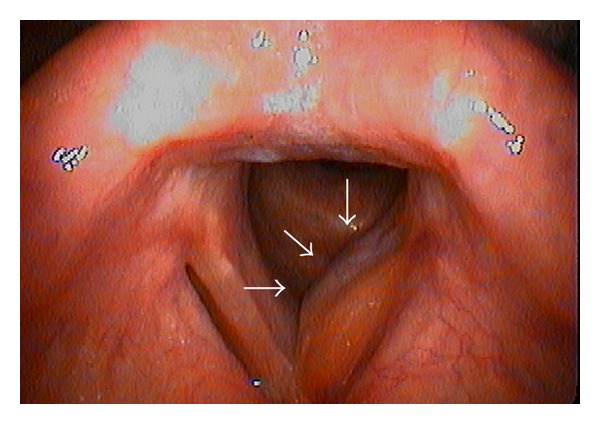
A submucosal swelling of the left subglottic trachea (arrow) by fiberscopic examination.

**Figure 2:**
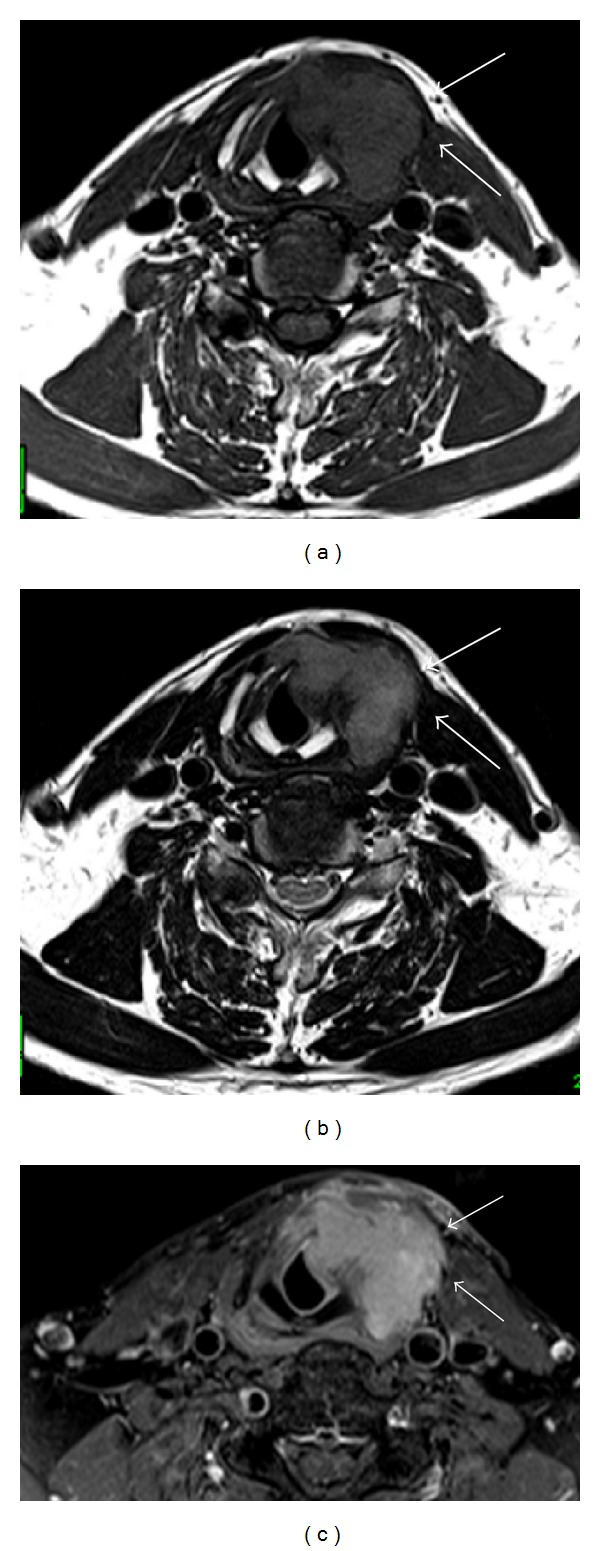
MRI revealed the same findings as CT scanning: (a) a low-intensity area on T1-weighted images and (b) a nonhomogenous high-intensity area on T2-weighted images. (c) The tumor demonstrates high intensity on a postgadolinium contrast T1 image (arrow).

**Figure 3:**
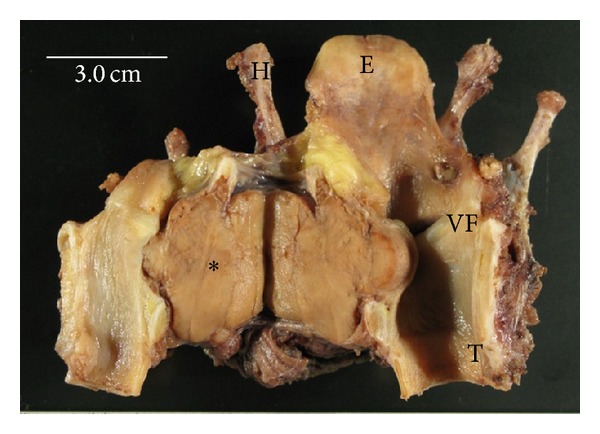
From behind, the laryngectomy specimen reveals a giant cell tumor under the intact mucosa (∗). E: epiglottis, H: hyoid bone, T: trachea, VF: vocal fold, and scale bar = 3.0 cm.

**Figure 4:**
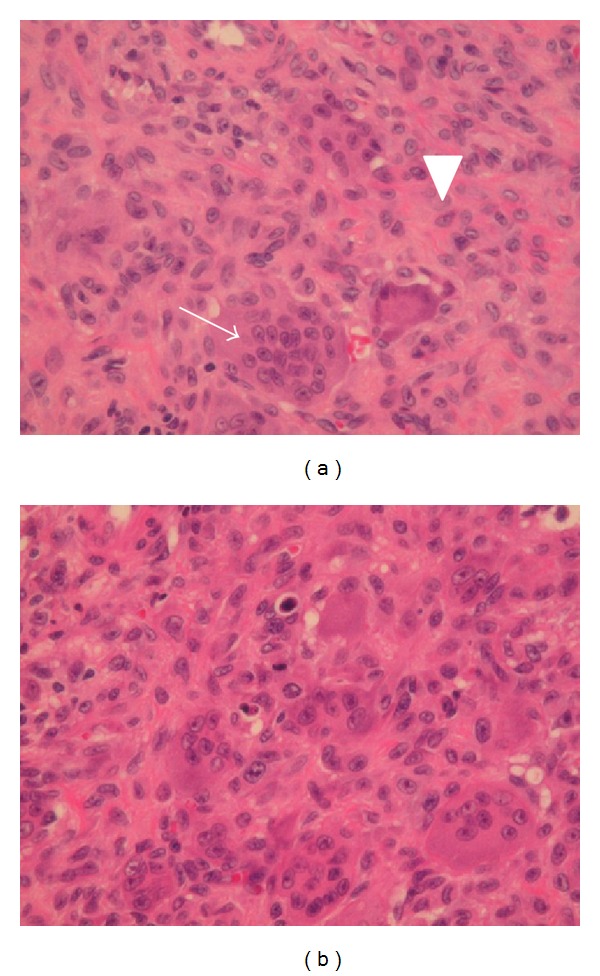
(a) Histological examination demonstrates that the cellular mononuclear eosinophilic stromal component (arrowhead) and multinucleated osteoclast-like giant cells (arrow) were scattered throughout the lesions in an intermediate power field. Pleomorphic giant cells are absent. (b) Mitotic figures are about 6/10 HPF by the lack of cytological atypia.

A 59-year-old man was referred to our department with a 3-month history of gradually worsening hoarseness and enlarging anterior neck mass. On physical examination, a firm and fixed mass measuring 3.0 cm at its maximum diameter was noted in the left region of the thyroid. Endoscopic examination revealed a submucosal swelling of his left vocal fold with intact movement. The laryngeal mucosa did not appear to have an ulcerative or hemorrhagic lesion (Figure 1). A computed tomography (CT) scan of his larynx showed cortical expansion and destruction at the left side of the thyroid cartilage. Magnetic resonance imaging (MRI) revealed the same findings as CT scanning: a low-intensity area on T1-weighted images (Figure 2(a)) and a nonhomogenous high-intensity area on T2-weighted images (Figure 2(b)). The images of the tumor demonstrated high intensity on a postgadolinium contrast T1 image (Figure 2(c)). Histopathological findings obtained by incisional biopsy supported a diagnosis of giant cell tumor. We performed total laryngectomy because the tumor involved more than half of the thyroid cartilage and cricoid. The surgical resected specimen, measuring 3.0 cm, showed an unencapsulated solid white and gray mass and was located under the intact mucosa. It destroyed the posterior lamina of the thyroid cartilage, but the anterior lamina was intact. The interface between the tumor and the adjacent mucosa and the cartilage was clearly delineated (Figure 3). Histological examination revealed a cellular mononuclear eosinophilic stromal component, and multinucleated osteoclast-like giant cells were scattered throughout the lesion in an intermediate power field (Figure 4(a)). Pleomorphic giant cells were absent. Mitotic figures were about 6/10 HPF by the lack of cytological atypia (Figure 4(b)). Histopathological findings yielded a diagnosis of “giant cell tumor with local invasion.” The patient has been well following total laryngectomy and subsequently free of disease for two and a half years.

## 3. Discussion

Giant cell tumors (GCTs) are relatively frequent skeletal tumors that occur mainly at the distal end of the femur and the proximal end of the tibia. In the head and neck region, GCTs are found mainly in the maxillary region [5] and at the base of the skull [6]. GCTs rarely arise from the cartilaginous laryngeal skeleton. The first case was published by Wessely in 1940 [4] and, after that study, a review of the literature uncovered 31 cases of GCTL ([4, 7–30], Table 1). Nishimura et al. reported [29] that the age at presentation averaged 41.7 years and ranged from 23 to 60 years. The male/female ratio was 9 : 1, whereas GCT of the bone is more frequent in women. The most typical clinical presentation, hoarseness and then anterior neck mass, does not differ substantially from that of other laryngeal malignancies. Radiologic investigations do not usually aid in differentiating GCT of the larynx from other neoplasms, so the diagnosis of GCT is established with an open biopsy.

The common head and neck sites for these tumors correspond to the cranial bones formed by endochondral ossification. The laryngeal skeleton is primarily composed of hyaline cartilage until approximately the second decade of life, at which time it may gradually begin to be replaced by bone. This occurs at different rates in different individuals but occurs earlier in males [26].

The treatment of GCTs is controversial, and no consensus exists on their management. A review of the literature reveals that the majority of patients were managed surgically (Table 1). Surgical excision of the tumor yielded excellent outcomes, but drawbacks include complications with voice quality. Bell et al. [31] advise radiotherapy. In this context, Rudert [13] has pointed out that a large proportion of radio-induced sarcomas were GCT of the bone, treated primarily with radiotherapy. It is difficult to estimate the risk of inducing a postirradiation sarcoma in GCT because of the small number of cases described in the literature. Radiation has been used as an adjuvant treatment in some cases, but the general consensus is that it is not needed and does not significantly affect outcome.

GCT of the bone is a locally osteolytic tumor with variable aggressiveness. In rare cases, pulmonary metastasis can be observed [32]. Additionally, Coyas et al. reported [30] that GCT of the larynx has been described as being malignant. The tumor appeared to arise from the soft tissue of the right vocal cord, and an osteocartilaginous origin was not documented. It is reported to have had pleomorphic histologic features. The tumor was characterized by multiple recurrences in spite of local excision and subsequent irradiation. It eventually involved the overlying skin. Although interpreted as a “metastasis,” it may actually have been soft tissue seeding. The patient has had no recurrence during 1 year of follow-up. This case is unusual with respect to its soft-tissue origin. However, even if it is accepted as a GCT of the soft tissue of the larynx, recurrence and implantation into adjacent soft tissue did not produce an adverse clinical outcome. If comparisons are drawn with GCT of the skeleton, similar clinical courses exist [33, 34].

## 4. Conclusion

GCT of the larynx is an uncommon entity, with very few cases reported in the literature. GCT of the larynx should be treated with complete surgical removal and has an excellent prognosis. We performed total laryngectomy on a patient with GCTL. The patient is free of recurrence after two and a half years, but long-term observation is still required.

## Figures and Tables

**Table 1:** Summary of reported cases of GCT of the larynx.

Case	Age	Sex	Location	Treatment	Follow-up
Wessely [4]	51	Male	Cricoid	RT	
Federova [7]	35	Male	Thyroid	Partial laryngectomy + RT	NED 1 y
Wagemann [8]	40	Male	Cricothyroid	Laryngectomy + RT	NED 7 y
Perrino [9]	32	Male	Cricoid	Laryngofissure + RT	NED 0.25 y
Kaliteevskii and Korol'kova [10]	52	Male	Thyroid	Laryngectomy	
Pohl [11]	50	Male	Thyroid	RT	NED 2 y
Kohn [12]	50	Male	Epiglottis	NA	
Rudert [13]	53	Male	Thyroid	Partial laryngectomy	NED 8 y
Hall-Jones [14]	26	Male	Thyroid	Laryngectomy	NED 1.5 y
Goto and Nakashima [15]	47	Male	Thyroid	Partial laryngectomy	
Kotarba and Niezabitowski [16]	60	Male	Epiglottis	Partial laryngectomy	NED 0.5 y
Ribari et al. [17]	35	Male	Cricoid	Laryngectomy + RT	NED 6 y
Kubo et al. [18]	40	Male	Cricoid	Laryngofissure + 5-Fluorouracil + RT	
Tsybyme and Bogdanskaia [19]	34	Male	Thyroid	Laryngofissure + RT	
Borghese et al. [20]	28	Male	Thyroid	Laryngofissure	NED 1.25 y
Badet et al. [21]	23	Female	NA	NA	
Murrell and Lantz [22]	42	Male	Thyroid	Laryngectomy + neck dissection	
Martin et al. [23]	23	Male	Thyroid	Partial laryngectomy	NED 5 y
Miyata et al. [24]	60	Male	Thyroid	Laryngectomy	
Werner et al. [25]	35	Male	Thyroid	Laryngectomy	NED 7.5 y
Hinni [26]	31	Male	Thyroid	Partial laryngectomy	NED 3 y
Wieneke et al. [27]	44	Male	Thyroid	Laryngectomy	NED 0.6 y
Wieneke et al. [27]	57	Male	Cricoid	RT	NED 1.6 y
Wieneke et al. [27]	37	Male	Cricoid	Partial laryngectomy	NED 13.9 y
Wieneke et al. [27]	40	Male	Thyroid	Partial laryngectomy	
Wieneke et al. [27]	53	Female	Thyroid	Partial laryngectomy	NED 16.9 y
Wieneke et al. [27]	62	Female	Thyroid	Laryngectomy	NED 24.9 y
Wieneke et al. [27]	26	Male	Thyroid	Laryngectomy	NED 1.5 y
Wieneke et al. [27]	37	Male	Thyroid	RT + cyclophosphamide	NED 19.4 y
Wong and Seikaly [28]	49	Male	Thyroid	Partial laryngectomy	NED 2.7 y
Nishimura et al. [29]	31	Male	Thyroid	Partial laryngectomy	NED 0.7 y
Coyas et al. [30]	67	Male	Vocal cord	Laryngofissure + RT	NED 1 y
Present case	59	Male	Thyroid	Laryngectomy	NED 2.5 y

NA: not available; NED: no evidence of disease; and RT: radiation therapy.
